# Investigation of the potential of *Brevibacillus* spp. for the biosynthesis of nonribosomally produced bioactive compounds by combination of genome mining with MALDI-TOF mass spectrometry

**DOI:** 10.3389/fmicb.2023.1286565

**Published:** 2023-12-14

**Authors:** Jennifer Jähne, Stefanie Herfort, Joerg Doellinger, Peter Lasch, Le Thi Thanh Tam, Rainer Borriss, Joachim Vater

**Affiliations:** ^1^Centre for Biological Threads and Special Pathogens, Proteomics and Spectroscopy (ZBS6), Robert Koch-Institute, Berlin, Germany; ^2^Division of Pathology and Phyto-Immunology, Plant Protection Research Institute (PPRI), Ha Noi, Vietnam; ^3^Institute of Marine Biotechnology e.V. (IMaB), Greifswald, Germany; ^4^Institute of Biology, Humboldt University Berlin, Berlin, Germany

**Keywords:** *Brevibacillus brevis*, parabrevis, schisleri, formosus, porteri, MALDI-TOF MS, genome mining, bioactive compounds

## Abstract

The biosynthetic potential of 11 *Brevibacillus* spp. strains was investigated by combination of genome mining with mass spectrometric analysis using MALDI-TOF mass spectrometry. These endophytic, plant associated *Brevibacillus* strains were isolated from crop plants, such as coffee and black pepper, in Vietnam. Draft genomes of these strains were available. They were classified (a) by comparison with type strains and a collection of genome-sequenced *Brevibacillus* spp. deposited in the NCBI data base as well as (b) by construction of a phylogenetic tree from the core sequences of publicly available genomes of Brevibacillus strains. They were identified as *Brevibacillus brevis* (1 strain); *parabrevis* (2 strains); *porteri* (3 strains); and 5 novel *Brevibacillus* genomospecies. Our work was specifically focused on the detection and characterization of nonribosomal peptides produced by these strains. Structural characterization of these compounds was performed by LIFT-MALDI-TOF/TOF mass spectrometric sequence analysis. The highlights of our work were the demonstration of the tyrocidines, a well-known family of cyclodecapeptides of great structural variability, as the main products of all investigated strains and the identification of a novel class of pentapeptides produced by *B. brevis*; *B. schisleri*; and *B. porteri* which we designate as brevipentins. Our biosynthetic studies demonstrate that knowledge of their biosynthetic capacity can efficiently assist classification of *Brevibacillus* species.

## Introduction

For maintenance of sustainable agriculture innovative measures are needed to preserve soils and to prevent reduction of biodiversity. These aims require the development and provision of environment-friendly biofertilizer and biocontrol agents against harmful phytopathogenic bacteria and fungi as well as nematocidal compounds. The usage of plant-associated endophytes and rhizobacteria living in symbiosis with plants has emerged as a promising strategy to contribute to these important goals. Such microorganisms attain increasing importance as efficient biofertilizers, biostimulants, and biocontrol agents as an environment-friendly alternative to conventional agriculture (Saharan and Nehra, 2011; Hardoim et al., 2015).

In particular, rhizobacteria interacting with plant roots in the rhizosphere and endophytes colonizing plant vessels and organs are able to enhance plant growth and to protect their plant host against deleterious effects of phytopathogenic competitors as well as against abiotic stress. Such organisms are qualified to increase plant harvest significantly and to protect plants against harmful bacteria, fungi, nematodes and insects. In addition, they are capable to elicit systemic resistance triggering plant defense mechanisms against plant diseases (Van Loon et al., 1998; Van Loon and Glick, 2004) and to mediate dynamic chemical communications between plants and microorganisms (Van Loon, 2007; Farag et al., 2013). In contrast to agrochemicals such biologicals are beneficial for the plant host, biodegradable and produced at site (Luchtenberg and Kamilowa, 2009). Such effects of endophytes and rhizobacteria depend on their biosynthetic potential (Mülner et al., 2020). Usually, they have the capacity to produce a wide spectrum of bioactive compounds both of ribosomal as well as nonribosomal origin, such as bioactive peptides, polyketides, lanthibiotics and bacteriocins which can be recruited for plant development and protection. Following these aspects of growth stimulation and plant protection our Vietnamese partners provided us with 110 endophytes and rhizobacteria isolated from crop plants, such as coffee and black pepper. They performed field trials to improve growth promotion and to use the biocontrol effects exerted by their isolates (Jähne et al., 2023; Tam et al., 2023).

Our role in this collaborative project was to characterize these plant protecting microorganisms and their secreted bioorganic compounds at the molecular level. In previous work as the first step draft genome sequences of the provided organisms from Vietnam were obtained (Tam et al., 2020). In a subsequent work these organisms were studied at the genomic and phylogenetic level to initiate exploration of their rich biosynthetic potential (Jähne et al., 2023; Tam et al., 2023). In the present study we now focus our efforts on investigating the ability of 11 endophytic *Brevibacillus* strains from Vietnam to produce bioactive compounds. This was achieved through a combination of MALDI-TOF mass spectrometry and extensive genome mining to clarify which of the compounds are not only predicted, but actually produced. This characterization approach opens up possibilities for understanding the biocontrol effects of the *Brevibacillus* isolates at the molecular level.

## Materials and methods

### Materials

α-cyanohydroxycinnamic acid (CCA) used as matrix for MALDI-TOF MS was obtained from Bruker (Bremen, Germany). Acetonitrile (ACN, HPLC-grade) was purchased from Merck (Darmstadt, Germany), trifluoroaceticacid (TFA) from Sigma-Aldrich (Deisenhofen, Germany). Brevibacillus type strains were available from DSM (Deutsche Sammlung von Mikroorganismen, Braunschweig, Germany).

### Cultivation of organisms

For the preparation of surface extracts the investigated *Brevibacillus* strains were grown on agar plates using either the Landy, the LB or the TSA medium as described previously (Mülner et al., 2020) solidified with 1.5% agar in petri dishes for 24; 48; and 72 h at 30°C. In addition, liquid fermentations were carried out in 100 ml Erlenmeyer flasks at 30°C to detect products released by the strains into the culture medium. Cells were harvested after 8; 10; 12; 24; 48; 72; and 96 h of incubation.

### Sample preparation

To study the biosynthetic potential of the investigated *Brevibacillus* strains their products were detected by MALDI-TOF MS: (a) in surface extracts of cells picked from agar plates or cell pellets harvested from liquid cultures by centrifugation for 10–20 min at 15000 rpm, (b) in culture supernatants after growth for 8; 10; 12; 24; 48; 72; and 96 h, and (c) after disintegration of cell material by solubilization with 80% trifluoroacetic acid.

### Profiling of bioactive compounds by MALDT-TOF MS

Bioactive compounds of the investigated *Brevibacillus* strains were detected and identified by MALDI-TOF MS, as outlined previously (Vater et al., 2018; Mülner et al., 2020). A Bruker Autoflex Speed TOF/TOF mass spectrometer (Bruker Daltonics; Bremen, Germany) was used with Smartbeam laser technology applying a 1 kHz frequency-triple Nd-YAG-laser (λ_ex_ = 355 nm). Samples (2 μl) of surface extracts and culture supernatants were mixed with 2 μl matrix solution (a saturated solution of α-hydroxy-cinnamic acid in 50% aqueous ACN containing 0.1% TFA) spotted on the target, air dried and measured. Mass spectra were obtained by positive-ion detection in reflector mode. Monoisotopic masses were observed. Parent ions were detected with a resolution of 10.000. Sequence analysis of peptide products was performed by MALDI-LIFT-TOF/TOF mass spectrometry in laser induction decay (LID) mode (Suckau et al., 2003). The product ions in the LIFT-TOF/TOF fragment spectra were obtained with a resolution of 1,000.

### Genome mining of Brevibacillus strains

Genome mining of the Brevibacillus strains isolated from crop plants in Vietnam and those which are deposited in the NCBI genome data base whose genomes have been sequenced were performed with antiSMASH 6.0 (Blin et al., 2021). Secondary metabolite gene clusters and their architecture were predicted comprising NRPS; PKS, hybrid PKS/NRPS; siderophores and bacteriocins. The results were correlated with mass spectrometric detection of the formed products by MALDI-TOF MS.

### Phylogenetic tree construction

An NGS-based core genome tree was constructed according to Na et al. (2018). For the genetic analysis of strains from the genus *Brevibacillus* we used a publicly available NGS dataset from NCBI (see Supplementary Table S1). The extraction of relevant core genome genes was done by the pipeline UBCG (up-to-date bacterial core gene) (Na et al., 2018). UBCG utilizes a set of 92 core genome genes calculated using complete genomes of 1.492 species covering 28 phyla.^1^ The UBCG pipeline was run using the default settings. Genomes were annotated using Prodigal v2.6.3 (Hyatt et al., 2010) and a homology search was done by Himmer3 v3.1b2.^2^ Hits were cut out, extracted genes were aligned by using Mafft v7.310 (Katoh and Standley, 2013) and then concatenated for filtering positions by multiple-sequence-alignments. Phylogenetic analysis was done by RAxML v8.2.12 (Stamatakis, 2014) with the UPGMA algorithm for hierarchical clustering. Finally, UBCG calculates the Gene Support Index (GSI) which indicates how many genes support the branch in the concatenated phylogenetic tree. The final tree was routed to the type strain *Paenibacillus polymyxa* DSM 36 and drawn using MEGA7 (Kumar et al., 2016).

### HPLC fractionation of the bioactive compounds

Amounts of 330 μl of surface and pellet extracts or culture filtrates of the investigated *Brevibacillus* strains were diluted with 660 μl 0.1% TFA, applied to a 300SB-C8-Zorbax column (4.6 × 250 mm) and fractionated by reversed-phase HPLC using an Agilent (1,200 series) instrument (Agilent Technology, Waldbronn, Germany). Bioactive compounds were eluted by a two-step gradient from 0 to 70% eluent B in 70 min and from 70 to 95% eluent B in 5 min (70–75 min) followed by isocratic elution at 95% eluent B for 10 min at a flow rate of 0.5 ml/min. Eluent A was 0.1% TFA in water, eluent B was 99.9% ACN/0.1% TFA. Fractions of 1 ml were collected and evaporated to dryness in a SpeedVac evaporator (Uniequip, Martinsried, Germany). The dried material was dissolved in 30 μl 50% aqueous ACN/0.1% TFA and analyzed mass spectrometrically.

## Results

Our research was focused on the characterization of 11 endophytic *Brevibacillus* strains which were isolated from crop plants, such as coffee and pepper, in Vietnam (Jähne et al., 2023). Draft genome sequences of these organisms have been obtained (Tam et al., 2020) which allow genome mining studies to detect gene clusters coding for the production of secondary metabolites, like NRPS; PKS; hybrids thereof; siderophores and bacteriocins, for example.

Characterization of these endophytic *Brevibacillus* strains according to their draft genome sequences was investigated by (a) phylogenetic analysis based on the UBCG pipeline and publicly available NGS datasets from *Brevibacillus* species deposited in the NCBI database, and (b) genome mining of *B. brevis*, *B. parabrevis*, *B. formosus*, and *B. porteri* strains also using NCBI genomes (see Table 1) exploring their biosynthetic potential.

**Table 1:** Genome mining of the 11 *Brevibacillus* strains from Vietnam in the context of type strains and genome sequenced, well known *Brevibacillus schisleri*; *brevis*; *parabrevis*; *formosus*; and *porteri* strains deposited in the NCBI data base indicating gene clusters coding for nonribosomal peptides (NRPS) and polyketides (trans-AT-PKS) as well as hybrids thereof (TransAT-PKS, NRPS).

		NRPS			Trans AT-PKS		TransAT-PKS, NRPS	
		Tyrocidine	Gramicidin	Brevipentin	Marthiapeptide	Macrobrevin	Aurantinin	Zwittermycin A
*Brevibacillus schisleri*	DSM30^T^	1.2	1.4	9.1			11.1	1.7
	NBRC100599	1.4	1.6/1.7	1.14			1.12	1.8
	LABIM17	1.4	1.6	1.13			1.11	1.7
	NRRL NRS 604	19.2	19.3/19.4	42.1			9.1	30.1/30.2
	NBRC15304	1.2	1.1	4.1			6.1	3.2
	NCTC2611	1.13	1.11	1.1			1.4	1.8
	G25-137	10.2	10.3	3.1			56.3	25.2
	ATCC35690	12.1	32.1/37.1	55.1			16.2	22.2
	BO11	1.4		1.12			1.1	1.6
*Brevibacillus brevis*	LDZV	1.4		1.13	1.6	1.14	1.11	1.7
	DZQ7	1.4		1.14	1.7	1.15	1.12	1.8
	HK544	1.9		1.16		1.15	1.3	1.7
	MS2.2	27.1		15.1	3.1	54.1/62.1	8.1	3.2
	Leaf182	36.1		17.1	37.1	17.2	30.1	37.2
*Brevibacillus parabrevis*	DSM8376^T^	29.1	7.2					
	CN1	47.1	56.1/83.1					
	NRRL NRS 605	21.1	6.1					
	NBRC12334	29.1	7.2/42.1					
	HD1.4A	10.1	1.3					
	HD3.3A	1.5	1.4					
*Brevibacillus formosus*	DSM9885^T^	2.2	2.4				1.6	1.2
	NRRL NRS863	9.1/13.2	3.1/9.2				1.1	3.2/30.1
	NBRC15716	3.2	3.4/33.2				18.1	12.1
	AF7	1.2				1.6	5.1	1.4
	NF2	1.5	1.6				1.13	1.7/1.9
	G25-125	3.1	3.3/37.7			37.4	37.1	37.6
*Brevibacillus porteri*	X23	1.4		1.14			1.11	1.7
	B-41110^T^	11.2		55.1			9.1	2.3
	W65	11.2		55.1			9.1/54.1	2.3
	HB1.1	2.1		11.1			1.6	1.2
	HB1.2	1.1		8.1			2.3	7.1
	HB1.4B	1.2		11.1			2.3	9.2
*Brevibacillus* Novel genomo species	HB2.2	1.2	1.1/24.1	4.1		6.1	18.1	30.1
	RS1.1	3.3	1.1/3.1			1.7	1.6	1.2
	HB1.3	1.2		9.1		1.1	3.1	10.2
	MS2.1A	1.5	1.6	1.13			1.11	1.7
	DP1.3A	1.13	1.12	1.3		1.2	1.6	1.10

**Figure 1:**
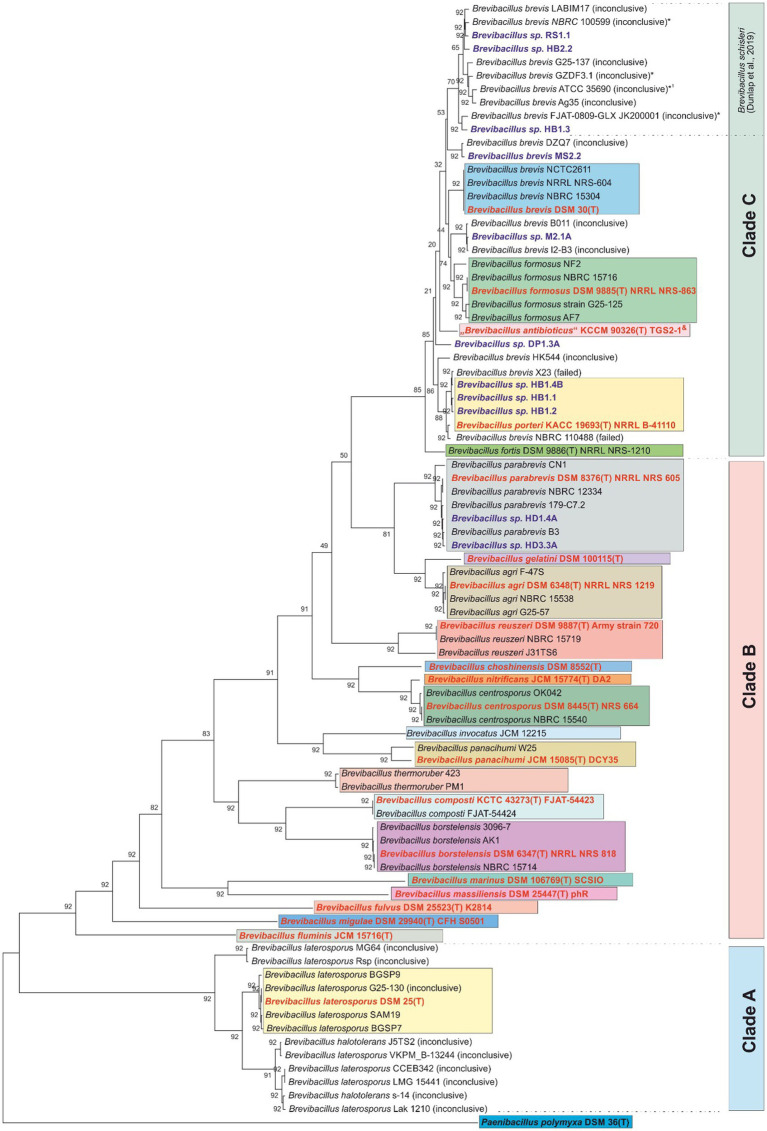
Phylogenetic tree based on 92 core genome genes (https://help.ezbiocloud.net/ubcg-gene-set/). The pipeline UBCGv3.0 (Na et al., 2018) was used to extract the nucleotide sequence of 92 core genome genes and a multiple-sequence-alignment was done by Mafft v7.310 (Katoh and Standley, 2013). To reconstruct their phylogeny RAxMLv8.2.12 (Stamatakis, 2014) was used with UPGMA algorithm. The Gene Support Index (GSI) which indicates how many genes support the branch in the concatenated phylogenetic tree is shown next to the branches. *Paenibacillus poymyxa* DSM 36^T^ was used as the outgroup. Color coding: red – type strains; dark blue – test strains of this study; blue – *B. brevis* group; green – *B. formosus* group; yellow – *B. porteri* group; grey – *B. parabrevis* group. “Inconclusive” or “failed”: results of the taxonomy check (species assignment not confirmed or failed).

To obtain a comprehensive and up-to-date picture of the taxonomic organization of the genus *Brevibacillus*, all available genomes of type strains and a selection of representative genomes from other strains of *Brevibacillus* were downloaded, whose genome mining data are summarized in Table 1. The phylogenetic tree constructed on the basis of 92 housekeeping genes from *Brevibacillus* genomes is shown in Figure 1. In this figure, bold red samples represent type strains, blue samples indicate plant protective bacteria isolated in Vietnam. Strains with the notation “inconclusive” or “failed” indicate the results of the standard NCBI taxonomy check. In Figure 1 differently colored boxes contain genomes obtained from type strains, and/or genomes of the same species that successfully passed the NCBI taxonomy check. Genomes with unclear, or failed check (denoted as “inconclusive” or “failed”) are not included in the boxes. For the phylogenetic analysis of Figure 1, the type strain of *Paenibacillus polymyxa* DSM 36^T^ was used as an outgroup.

The tree given in Figure 1 distinguishes three major clades A-C each containing distinct *Brevibacillus* species. First, it is striking that the taxonomic tree does not contain any unassignable strains in clade B (see, for example, strains of the species *B. parabrevis*, *B. gelatini*, *B. agri*, *B. reuszeri*, and others up to *B. fluminis*). On the other hand, clade A and C contain many genomes from isolates whose species assignment was unconfirmed by the NCBI taxonomy check. This concerns numerous strains originally assigned as *B. laterosporus* (see clade A), but, in particular, also many isolates assigned as *B. brevis*, *B. formosus*, *B. schisleri*, and *B. porteri* (see clade C).

In the context of the present study, most relevant results of this phylogenetic analysis are confirmations of species assignments of some of the endophytic *Brevibacillus* strains from Vietnam according to our previous report (Jähne et al., 2023). For example, the isolates *Brevibacillus* sp. HB1.4B, HB1.1, and HB1.2 are found in the cluster of the type strain of *Brevibacillus porteri*, while the isolates *Brevibacillus* sp. HD1.4A and HD3.3A can be mapped analogously to *Brevibacillus parabrevis*. In the upper part of clade C, the tree shows a larger cluster of a total of 10 genomes named *Brevibacillus brevis* with inconclusive species assignment (NCBI), and 3 novel genomospecies from plant protective isolates (Brevibacillus RS1.1, HB2.2, HB1.3) (Jähne et al., 2023). In 2019 Johnson and Dunlap assigned some of these genomes to the new species *Brevibacillus schisleri*, with the proposed type strain *B. schisleri* ATCC 35690 (marked with *1 in Figure 1). The results of this phylogenetic analysis confirm the results and conclusions of previous studies by others (Johnson and Dunlap, 2019) and by our groups (Jähne et al., 2023). Species assignment for the other *Brevibacillus* sp. MS2.1A and DP1.3A is not possible by UBCG analysis alone. However, these strains were assigned by Jähne et al. (2023) as members of novel genomospecies proposed to exist in clade A.

Genome mining studies with these strains were performed with antiSMASH 6.0 (Blin et al., 2021). The obtained results were summarized in Table 1. Seven representative gene clusters were detected coding for the production of the nonribosomal peptides: the tyrocidines (Tang et al., 1992; Hitzeroth et al., 2005) and gramicidins (Tang et al., 1992; Govaerts et al., 2001; Chitta and Gross, 2004; Kessler et al., 2004), for the polyketide macrobrevin (Helfrich et al., 2018; Eltokhy et al., 2021; Chakraborty et al., 2022), for the marthiapeptide (Zhou et al., 2012) as well as for aurantinin- (Nishikori et al., 1978; Yang et al., 2016; Li et al., 2021) and zwittermycin A-like compounds (Emmert et al., 2004; Kevany et al., 2009). In addition, a gene cluster was found which codes for the biosynthesis of a hitherto unknown family of pentapeptides which we have detected and structurally characterized mass spectrometrically, as will be outlined in detail later. These novel compounds we designate brevipentins.

From Table 1 it is apparent that tyrocidine biosynthetic genes were found in all investigated *Brevibacillus* species, while those coding for gramicidin biosynthesis are present only in the genomes of *B. schisleri*, *B. parabrevis*, and *B. formosus* strains as well as in the genomes of the novel *Brevibacillus* genomospecies HB2.2, RS1.1, MS2.1A, and DP1.3A. Gene clusters coding for the formation of brevipentins were detected in the genomes of *B. schisleri*, *B. brevis*, and *B. porteri* strains, but not in *B. parabrevis* and *B. formosus*. Macrobrevin biosynthetic genes were exhibited specifically by *B. brevis* and *B. formosus* strains as well as by the novel *Brevibacillus* genomospecies. Gene clusters for aurantinin- and zwittermycin-like compounds were found in the genomes of all investigated Brevibacillus species with exception of *B. parabrevis*. These features were demonstrated by genome mining studies with *Brevibacillus* strains whose genome sequences are deposited in the NCBI data base, as demonstrated in Table 1.

However, the biosynthetic capacity of the investigated *Brevibacillus* strains can only be determined by isolation and structural analysis of the actually produced compounds. For this important task mass spectrometry is the method of choice because of its unsurpassed sensitivity and resolution. In our work we used MALDI-TOF MS to detect and characterize the products of the endophytic strains from Vietnam and of selected type strains for comparison. Secondary metabolite production was investigated in detail for the following representative test strains: *B. brevis* MS2.2; *B. parabrevis* HD3.3A; *Brevibacillus* sp. HB2.2 and *B. porteri* HB1.1. Product formation by these strains was studied in a time and space dependent manner. *Brevibacillus* strains were grown in three different cultivation media (Landy; LB and TSA medium) both on agar plates as well as in liquid cultures. Cell material was picked from agar plates at 24; 48; and 72 h. Samples from liquid cultures were taken at 8; 10; 12; 24; 48; 72; and 96 h. Surface extracts were prepared from cells picked from agar plates or cell pellets obtained by centrifugation of liquid cultures by extraction with 50% acetonitrile (ACN) containing 0.1% trifluoroacetic acid (TFA).

**Figure 2:**
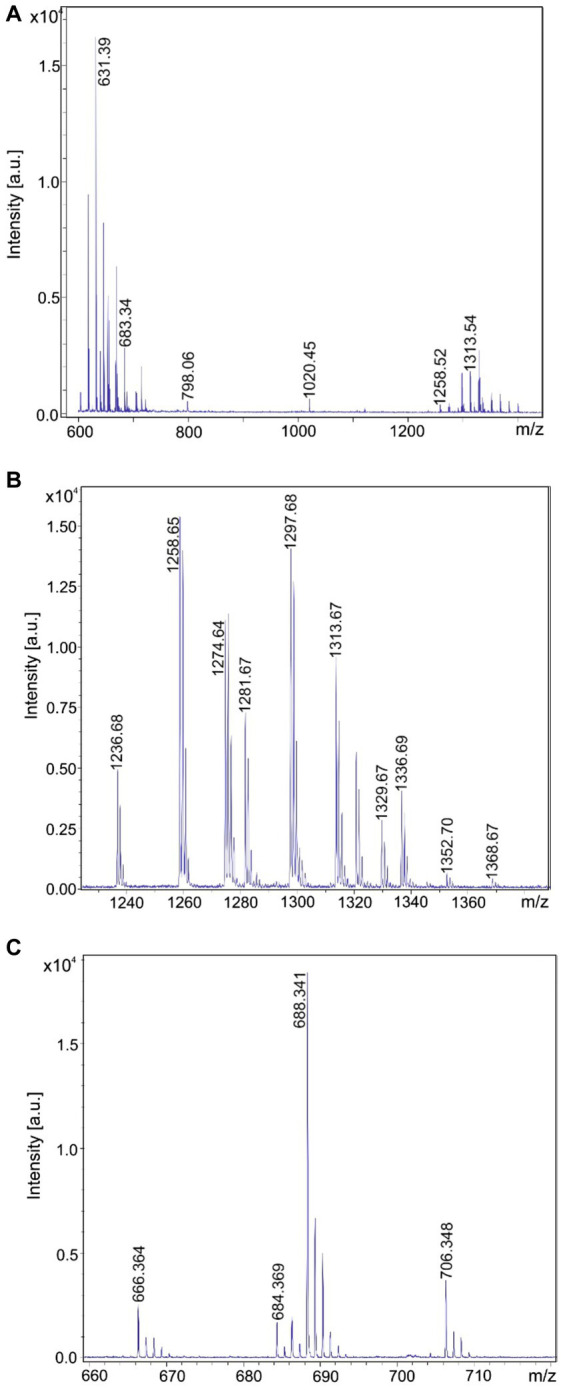
Detection of tyrocidines, gramicidins and the marthiapeptide produced by *Brevibacillus brevis* strain MS2.2. **(A)** MALDI-TOF mass spectrum of a surface extract of strain MS2.2 grown on agar plates using the Landy medium for 48 h in the mass range from *m/z* = 600–1,500. **(B)** Mass spectrum of the produced tyrocidine species in the mass range *m/z* = 1,200–1,400. **(C)** Mass spectrum of the marthiapeptide in the range of m/z = 660–720.

**Figure 3:**
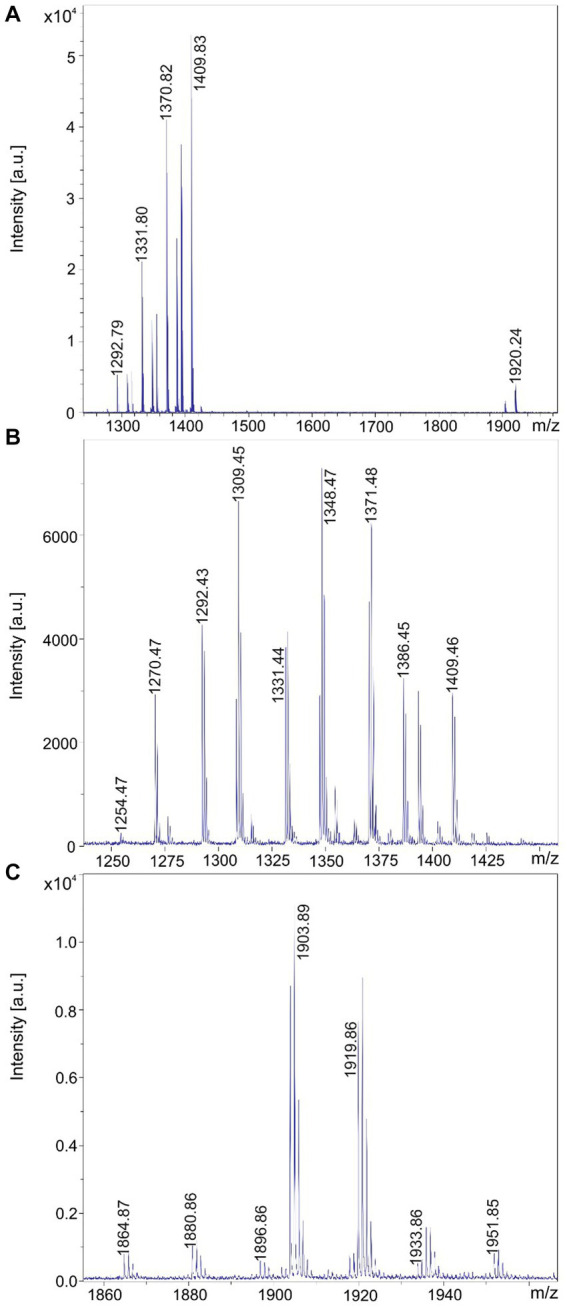
Detection of tyrocidines and C15-gramicidins produced by *Brevibacillus parabrevis* strain HD3.3A. **(A)** MALDI-TOF mass spectrum of a surface extract of strain HD3.3A grown on agar plates using the Landy medium for 48 h in the mass range from *m/z* = 1,300–2000. **(B)** Mass spectrum of the produced tyrocidine species in the mass range *m/z* = 1,200–1,400. **(C)** Mass spectrum of gramicidins detected in the mass range of *m/z* = 1850–1960.

**Figure 4:**
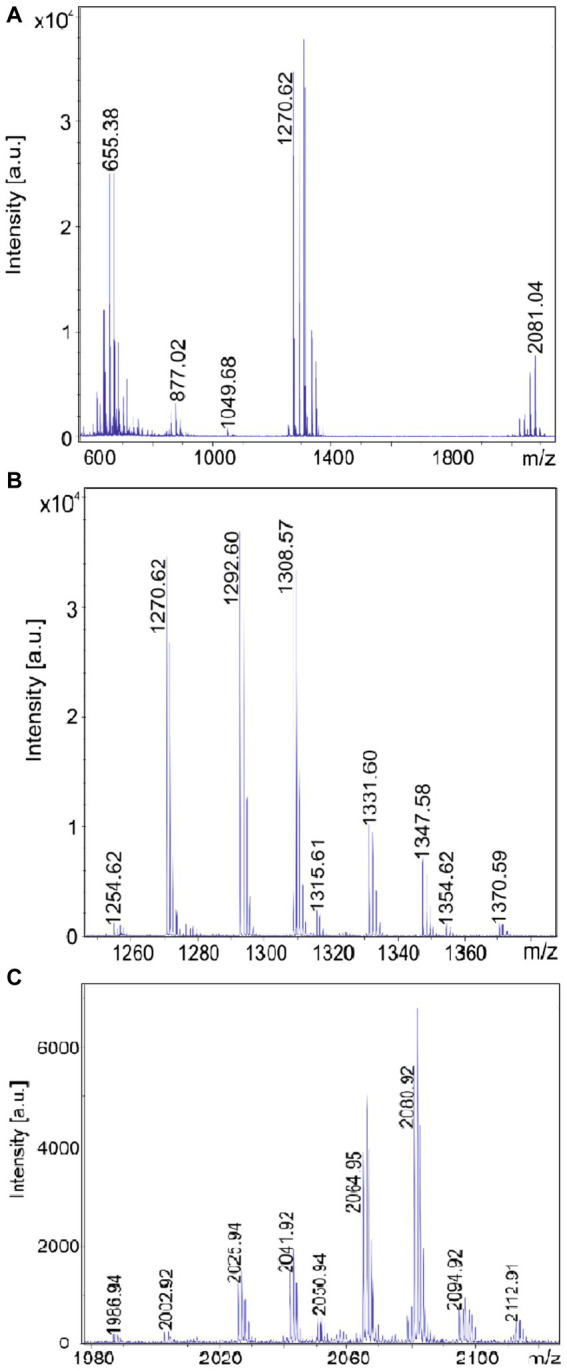
Detection of tyrocidines and C17-gramicidins produced by *Brevibacillus* sp. strain HB2.2. **(A)** MALDI-TOF mass spectrum of a surface extract of strain HB2.2 grown on agar plates using the Landy medium for 48 h in the mass range from *m/z* = 600–2000. **(B)** Mass spectrum of the produced tyrocidine species in the mass range *m/z* = 1,250–1,380. **(C)** Mass spectrum of gramicidins detected in the mass range of *m/z* = 1980–2,200.

**Figure 5:**
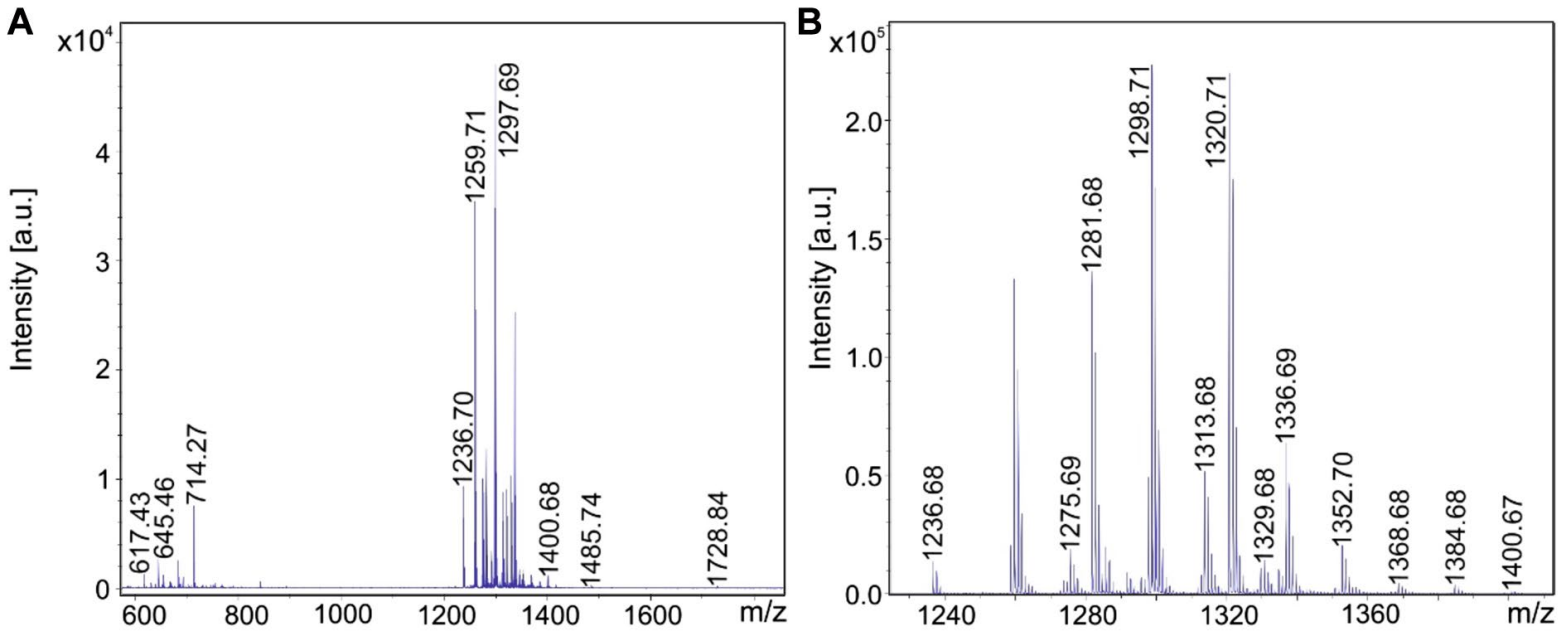
Detection of tyrocidines produced by *Brevibacillus porteri* strain HB1.1. **(A)** MALDI-TOF mass spectrum of a surface extract of strain HB1.1 grown on agar plates using the Landy medium for 48 h in the mass range from *m/z* = 600–1800. **(B)** Mass spectrum of the produced tyrocidine species in the mass range *m/z* = 1,230–1,400.

MALDI-TOF mass spectra of the products formed by the test strains are presented in Figures 2–5. In Figures 2A–5A MALDI-TOF mass spectra of surface extracts of strains *B. brevis* MS2.2, *B. parabrevis* HD3.3A, *Brevibacillus* sp. HB2.2 and *B. porteri* HB1.1 are shown in the mass range of *m/z* = 600–2,000 giving an overview on the bioactive products formed by these organisms. The tyrocidines are the dominant products of all *Brevibacillus* strains listed in Table 1, as demonstrated for *B. brevis* MS2.2, *B. parabrevis* HD3.3A*; Brevibacillus* sp. HB2.2 and *B. porteri* HB1.1 in Figures 2B–5B. Tyrocidines are a well-known family of cyclodecapeptides of high structural variability (Tang et al., 1992; Hitzeroth et al., 2005). Genome mining with antiSMASH 6.0 showed that in the genomes of all these strains the gene cluster coding for tyrocidine synthetase appears contiguously at one gene locus. The patterns of the formed tyrocidine variants are specific for each *Brevibacillus* species which will be described and discussed in detail in a forthcoming paper (Herfort et al., in preparation).

*B. schisleri*, *B. parabrevis*, *B. formosus* strains and the novel genomospecies produce gramicidins which also show structural variety (Tang et al., 1992; Govaerts et al., 2001). In Figure 3C the main gramicidin products for *B. parabrevis* HD3.3A was detected at mass numbers [M + H, Na; K]^+^ = 1881.9; 1903.9; and 1919.9 Da. The gramicidins formed by *Brevibacillus* sp. HB2.2 appeared at higher mass numbers. The main component was found at [M + H, Na; K]^+^ = 2042.9; 2064.9; and 2080.9 in Figure 4C.

In addition, *B. brevis* strain MS2.2 forms the marthiapeptide indicated by mass numbers [M + H, Na; K]^+^ = 666.4; 688.3; and 704.3 (Figure 2C). All the products detected mass spectrometrically in Figures 2–5 represent nonribosomal peptides which are located at the surface of *Brevibacillus* cells, as shown in Supplementary Figure S1 for *Brevibacillus* sp. HB2.2 and *B. parabrevis* HD3.3A. They were found in surface extracts of cells picked from agar plates as well as those obtained from liquid cultures, but not in culture filtrates.

**Figure 6:**
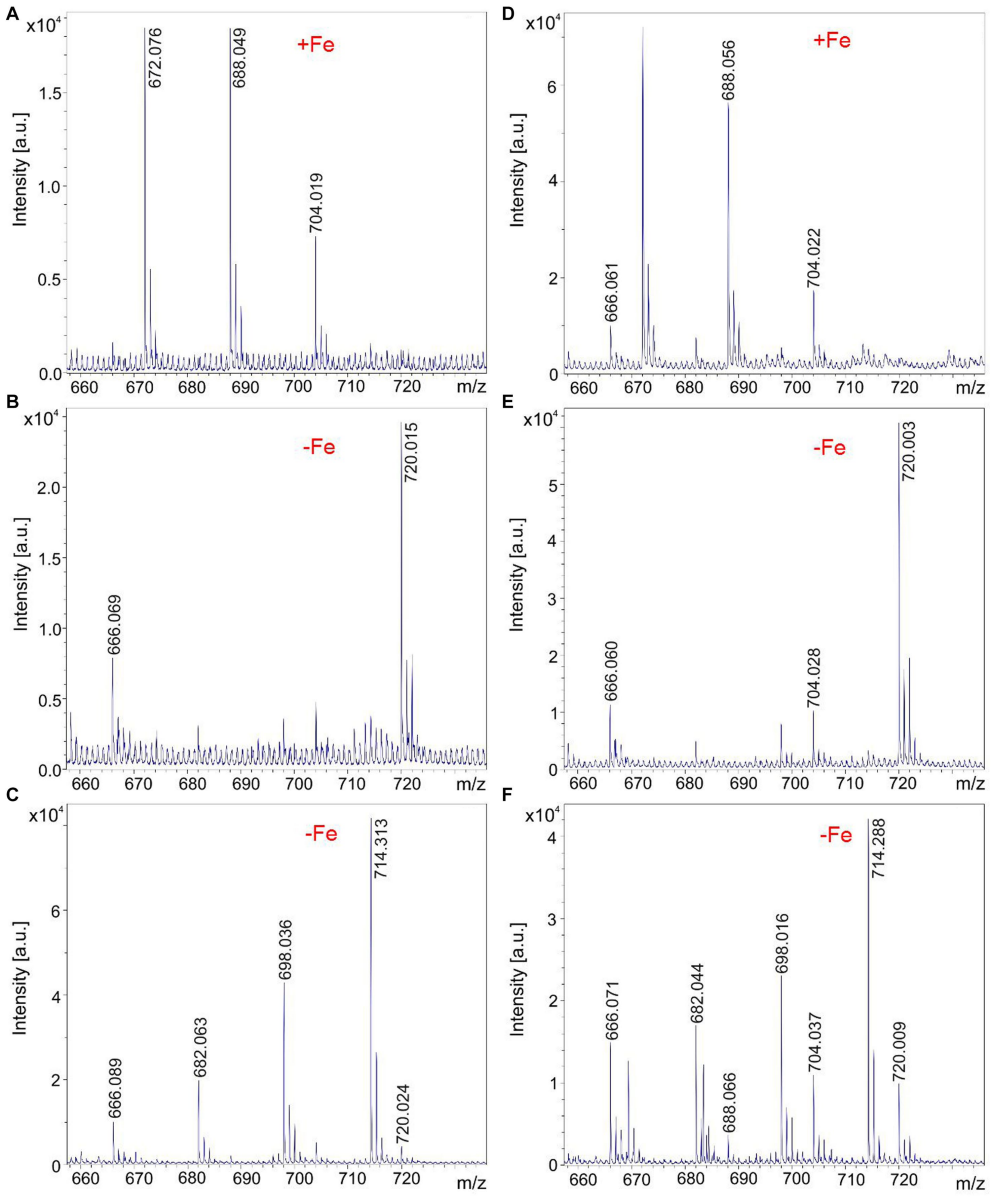
Production of the siderophore petrobactin by *Brevibacillus parabrevis* HD3.3A **(A–C)** and *Brevibacillus* sp. HB2.2 **(D–F)** grown in the Landy medium for 48 h. **(A–C)** MALDI-TOF mass spectra of culture filtrates of HD3.3A grown in the presence **(A)** and after depletion of iron **(B)**. **(C)** Mass spectrum of a surface extract of HD3.3A cells grown in the absence of iron detected in the mass range of *m/z* = 660–730. **(D,E)** MALDI-TOF mass spectra of culture filtrates of HB2.2 grown in the presence **(D)** and after depletion of iron **(E)**. **(F)** Mass spectrum of a surface extract of HB2.2 cells grown in the absence of iron detected in the mass range of *m/z* = 660–730.

All investigated endophytic *Brevibacillus* strains isolated from crop plants in Vietnam form the siderophore petrobactin which is efficiently released into the culture medium specifically on iron depletion, as demonstrated in Figure 6 for *B. parabrevis* HD3.3A (Figures 6A–C) and *Brevibacillus* sp. HB2.2 (Figures 6D–F). Petrobactin was detected by MALDI-TOF MS at *m/z* = 720.0. Most of the siderophore was found in the culture medium, but a minor amount of petrobactin was observed in surface extracts of these strains, too.

In addition, for *B. brevis* and *B. porteri* strains as well as for the novel strains HB2.2, RS1.1, MS2.1A, and DP1.3A yet unknown compounds with mass numbers of 984.4; 1002.5; and1020.6 Da were detected, as shown for *Brevibacillus* sp. HB2.2 in Supplementary Figure S2. Also, for *B. porteri* strain HB1.1 unknown products with mass numbers of 1673.0; 1729.0; 1772.0; and 1829.0 Da were observed (Supplementary Figure S3). All of them have still to be identified. The polyketide macrobrevin as well as the aurantinin and zwittermycin A-like compounds derived from genome mining in Table 1 have not yet been detected by MALDI-TOF mass spectrometry.

**Figure 7:**
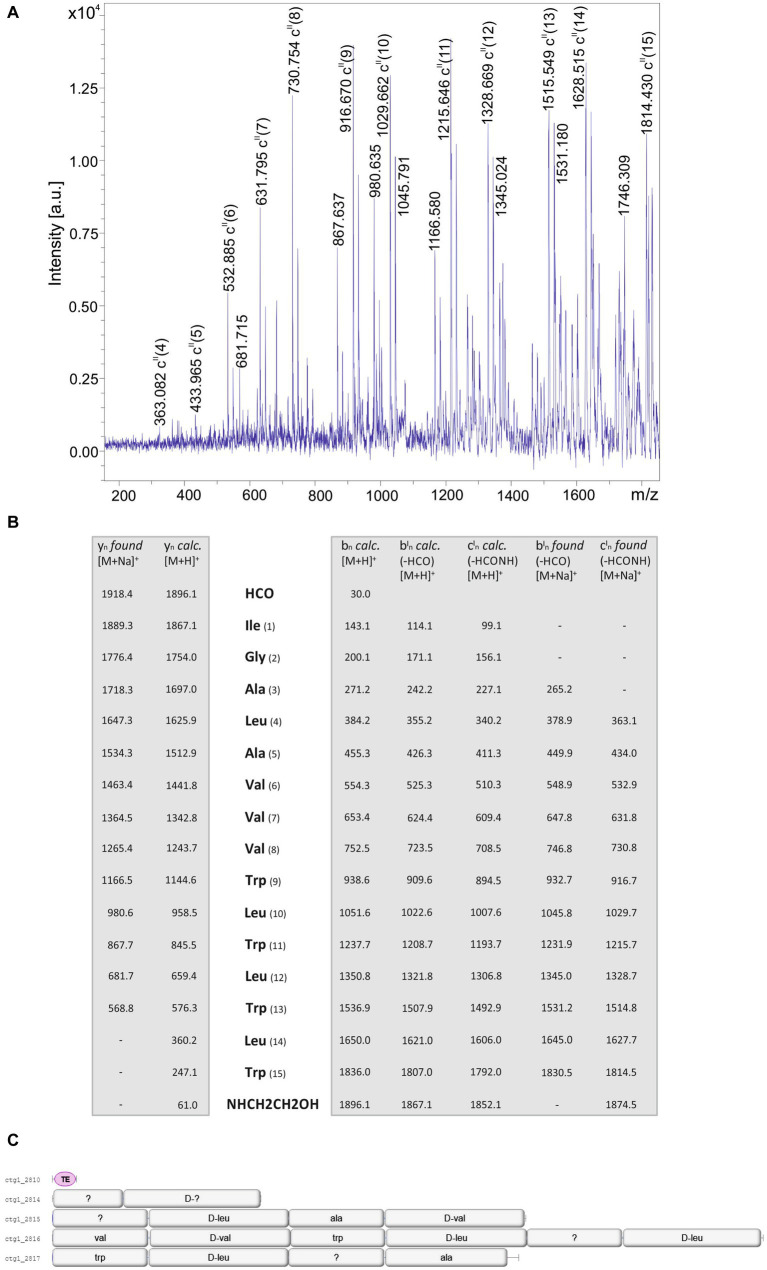
**(A)** LIFT-MALDI-TOF/TOF product ion spectrum of a C15 gramicidin produced by *Brevibacillus parabrevis* HD3.3A with a molecular mass of *m/z* = 1896.1. **(B)** Mass spectrometric sequence determination of this gramicidin species derived from the product ion pattern obtained by the LIFT-MALDI-TOF/TOF fragment spectrum shown above. **(C)** Module organization of the C15-gramicidin multienzyme system derived from genome mining of *Brevibacillus parabrevis* HD3.3A by AntiSMASH 6.0. The modules are labeled with their substrate amino acids activated at their reaction centers to correlate module arrangement with the sequence of the peptide product which are colinear.

Structural analysis of the products of the investigated *Brevibacillus* strains was initiated by mass spectrometric fragment analysis using LIFT-MALDI-TOF/TOF MS. In Figure 7 the sequence determination of a gramicidin product of *B. parabrevis* HD3.3A (parent ion: [M + H]^+^ = 1896.1 Da) is demonstrated which was derived from the LIFT-MALDI-TOF/TOF fragment spectrum shown in Figure 7A. Fragment analysis confirmed the sequence of a well-known C15-gramicidin formerly reported by other authors (Tang et al., 1992; Govaerts et al., 2001; Chitta and Gross, 2004; Kessler et al., 2004). Linear gramicidins are modified both at their N- and C- terminus by a formyl residue and ethanolamine, respectively. The results of mass spectrometric sequencing of this gramicidin variant exhibited in Figure 7B shows that sequence analysis can be based on y_n_-fragment ions as well as b_n_- and c_n_- fragment ions obtained on loss of the N-terminal formyl residue. In each case sodium adducts of these fragment ions were detected. B_n_-ions of the intact gramicidin compound were not found. Genome mining of the two *B. parabrevis* strains HD3.3A and HD1.4A showed (Figure 7C) that here the complete gene cluster is located on one single gene locus coding for gramicidin synthetase, a multienzyme complex, organized into four multifunctional enzymes composed of 2; 4; 6; and 4 modules, respectively.

**Figure 8:**
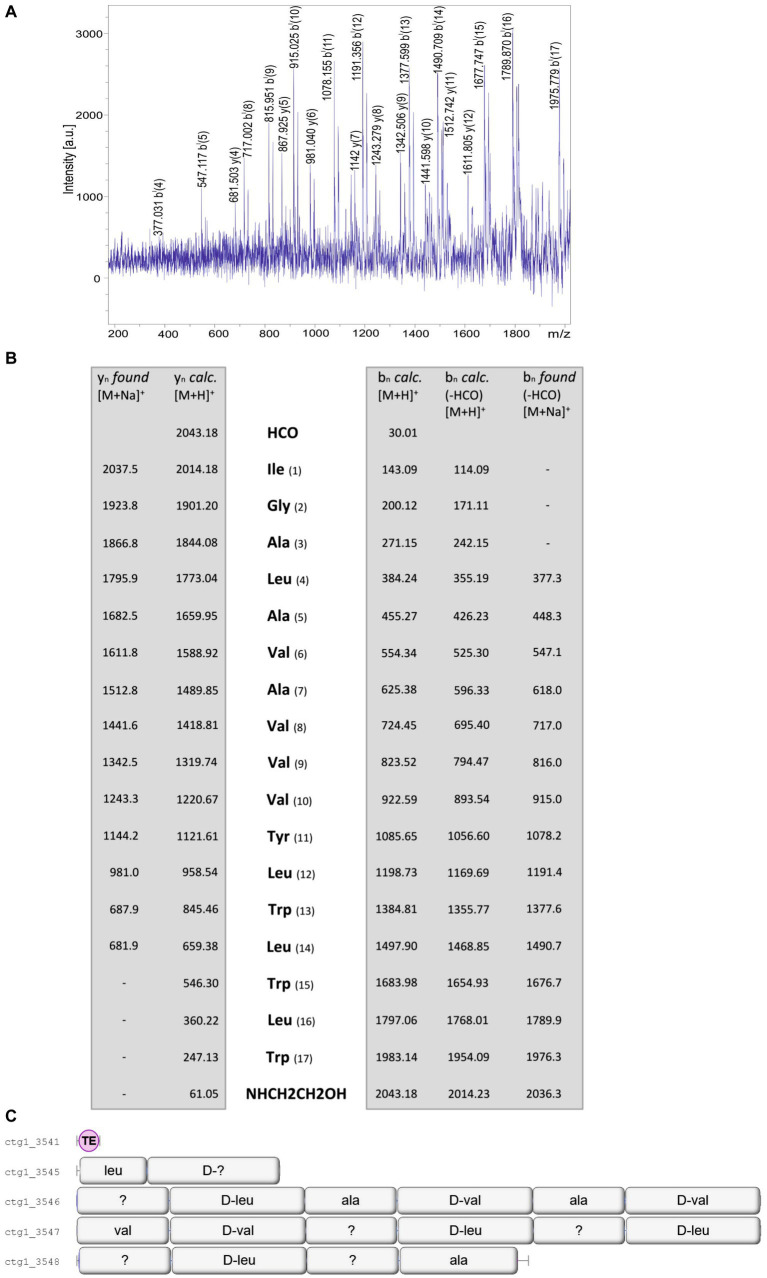
**(A)** LIFT-MALDI-TOF/TOF product ion spectrum of a C17-gramicidin produced by Brevibacillus sp. HB2.2 with a molecular mass of m/z = 2043.2. **(B)** Mass spectrometric sequence determination of this gramicidin species derived from the product ion pattern obtained by the LIFT-MALDI-TOF/TOF fragment spectrum shown above. **(C)** Module organization of the C17-gramicidin multienzyme system derived from genome mining of *Brevibacillus* sp. HB2.2 by AntiSMASH 6.0. The modules are labeled with their substrate amino acids activated at their reaction centers to correlate module arrangement with the sequence of the peptide product which are colinear.

More complex patterns of organization were found for the gramicidins produced by the investigated *B. schisleri* and the novel *Brevibacillus* sp. strains. Figure 8 shows the mass spectrometric sequence determination of a gramicidin species produced by *Brevibacillus* sp. HB2.2 with a parent ion [M + H]^+^ = 2043.1 Da derived from the product ion pattern obtained by LIFT-MALDI-TOF/TOF fragment ion spectrum depicted in Figure 8A. The same result was obtained for *Brevibacillus* sp. DP1.3A. In contrast to the C15-gramicidins found for *B. parabrevis* these strains form C17-gramicidins (Hansen et al., 2020), as demonstrated in Figure 8B. Here sequence analysis of the C17-gramicidin was based on the sodium adducts both of y_n_-fragment ions and b_n_-ions obtained after loss of the N-terminal formyl residue. For strains HB2.2 and DP1.3A the complete gene cluster coding for the gramicidin synthetase is located on one single gene locus apparent from Figure 8C. Here the corresponding multienzyme system is organized into four multifunctional enzymes composed of 2; 6; 6 and 4 amino activating modules, respectively. C17-gramicidins were reported for the first time by Hansen et al. (2020) and designated as britacidins.

Genome mining for the other novel *Brevibacillus* strains indicate that RS1.1 and MS2.1A also contain gramicidin biosynthetic genes, but here the whole gene cluster was truncated and the genes are spread in several contigs representing the draft genomes. For RS1.1 only 16 and for MS2.1A only 8 of the expected 18 modules of gramicidin synthetase were detected. In the genome of strain HB1.3 gramicidin biosynthetic genes were completely missing. As the consequence, these *Brevibacillus* strains do not produce gramicidin.

**Figure 9:**
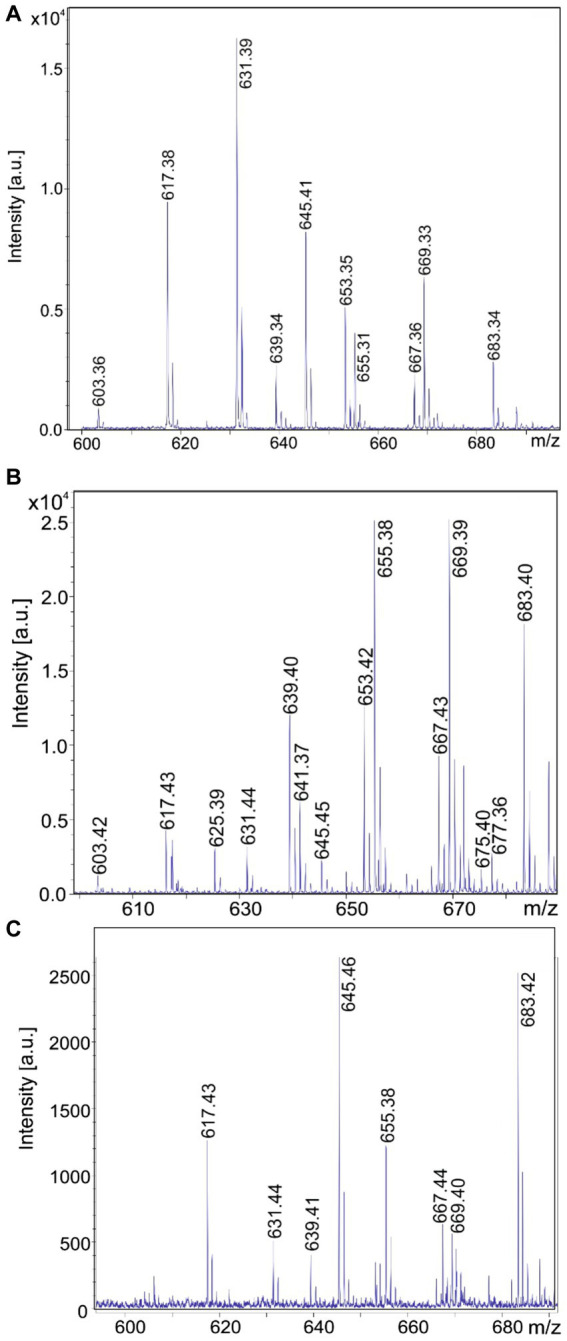
MALDI-TOF mass spectra of brevipentins, a novel class of pentapeptides produced by *Brevibacillus brevis* MS2.2 **(A)**; *Brevibacillus* sp. HB2.2 **(B)** and *Brevibacillus porteri* HB1.1 **(C)**. These products were detected in surface extracts of these strains grown on agar plates using the Landy medium for 48 h.

**Figure 10:**
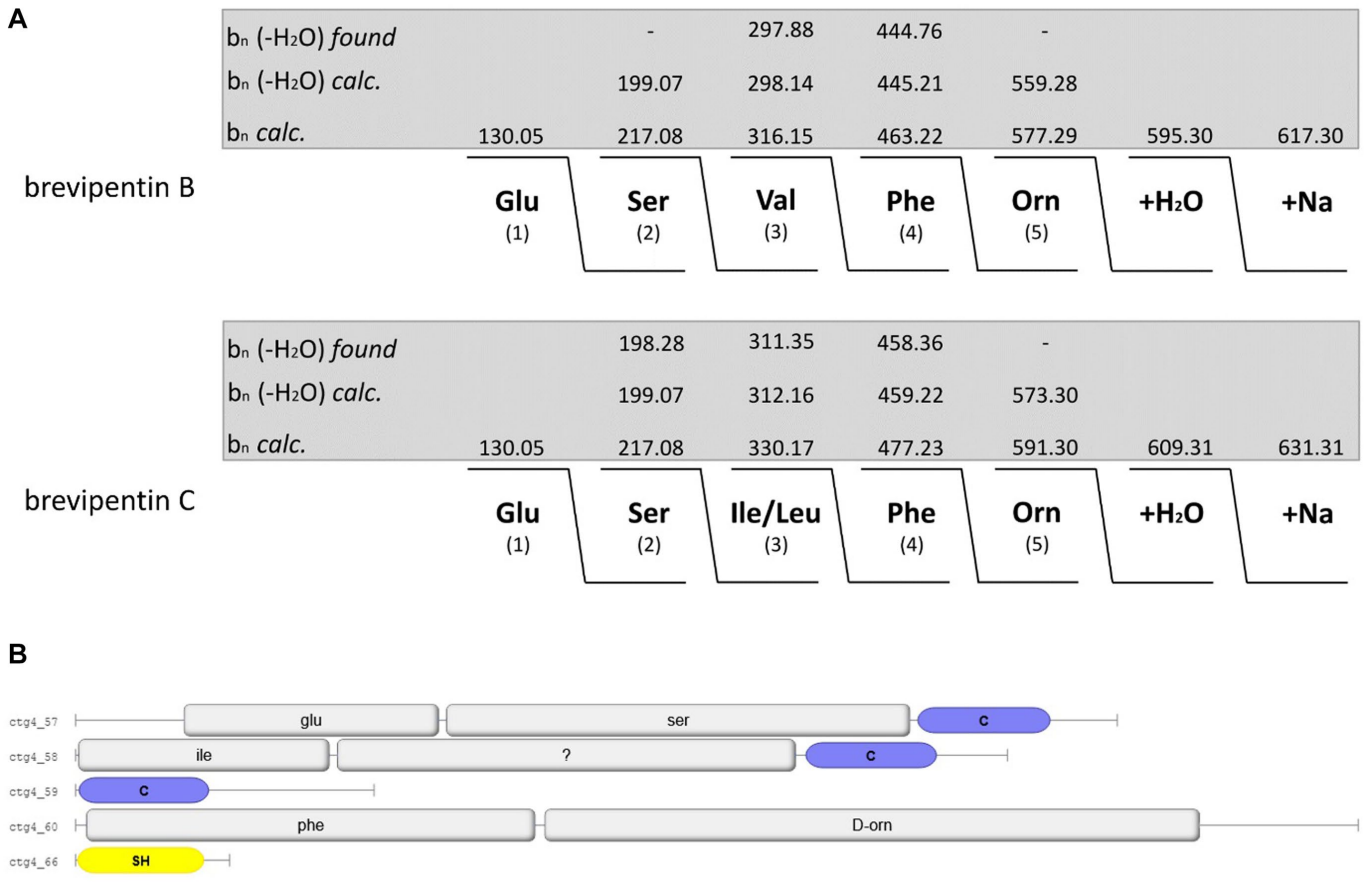
**(A)** Mass spectrometric sequence determination of brevipentins B and C produced by *Brevibacillus brevis* MS2.2 with parent ions [M + Na]^+^ = 617.7 and 631.7 Da from product ion pattern obtained by LIFT-MALDI-TOF/TOF fragment spectra. **(B)** Module organization of the brevipentin multienzyme system derived from genome mining of *Brevibacillus brevis* MS2.2.

In Figures 9A–C a family of yet unknown compounds was detected by MALDI-TOF MS at mass numbers of *m/z* = 603.3; 617.3; 631.3; and 645.3 which are specifically produced by *B. schisleri*, *B. brevis*, and *B. porteri* strains, but not by *B. parabrevis*. They were detected in surface extracts of the producer strains indicating that they are attached to the outer surface of these organisms. Also, the novel genomospecies with the exception of strain HB1.3 produced these agents. Structural investigation of these products was initiated by LIFT-MALDI-TOF/TOF fragment analysis. In Figure 10 the results were exhibited for the species found at *m/z* = 617.3 and 631.3 produced by *B. brevis* strain MS2.2. Sequence analysis indicated the formation of novel pentapeptides X-Y-Z-Phe-Orn which we designate brevipentins. They always appeared as sodium adducts (X = Glu or Asp; Y = Ser or Thr; Z = Val or Ile/Leu). The Phe and Orn components are strictly conserved in brevipentins. Their structure together with the detected alkali adducts are summarized in Table 2. The biosynthesis of these compounds can be attributed to the gene cluster shown in Figure 10B for strain *B. brevis* MS2.2. AntiSMASH-6.0 analysis indicates homology of this cluster with pacidamycin biosynthesis (Chen et al., 1989; Grüschow et al., 2009; Rackham et al., 2010; Zhang et al., 2010). However, there is no relationship among the structural genes coding for the brevipentin multienzme system with pacidamycin biosynthetic genes. Presumable, the homology implied by genome mining refers to peripheral function units. According to Figure 10B brevipentin synthetase is a complex of three multifunctional enzymes. Enzyme 1 activates Glu and Ser, while enzyme 3 uses Phe and Orn as substrates. Enzyme 2 is composed either of two or three modules. Two modules were observed for *B. brevis* MS2.2 and *Brevibacillus* sp. HB2.2, while three modules were found for *Brevibacillus* sp. HB1.3; M2.1A and DP1.3A as well as for *B. porteri* strains HB1.1; HB1.2; and HB1.4B. One module of brevipentin synthetase 2 activates hydrophobic amino acids, either Val or Ile/Leu, while the function of the other two modules remains unclear. The structure of the brevipentin products implies that both modules are not used in the biosynthetic process. This interesting point has to be clarified in more detail.

**Table 2:** Brevipentins produced *by Brevibacillus brevis, B. schisleri*; and *B. porteri.*

	[M + Na]^+^	[M + K]^+^	[M − H + 2Na]^+^	[M − H + Na, K]^+^	Structure
Brevipentin A	603.3	619.3	625.3	641.3	Asp-Ser-Val-Phe-Orn
Brevipentin B	617.3	633.3	639. 3	655.3	Glu-Ser-Val-Phe-Orn
Brevipentin C	631.3	647.3	653.3	669.3	Glu-Ser-Ile/Leu-Phe-Orn
Brevipentin D	645.3	661.3	667.3	683.3	Glu-Thr-Ile/Leu-Phe-Orn

**Figure 11:**
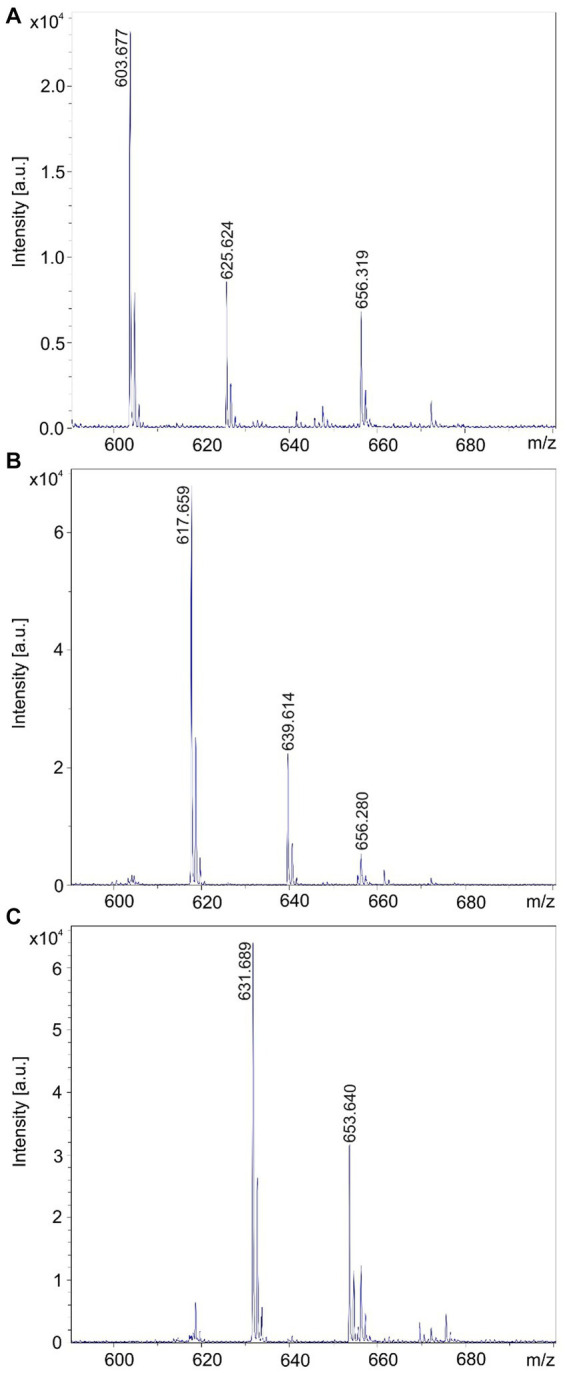
Separation of the brevipentin complex by RP-HPLC of an extract of the cell pellet obtained by growing *Brevibacillus* sp. HB2.2 in the Landy medium for 48 h. **(A-C)** Brevipentins A, B, and C with mass numbers of *m/z* = 603.7; 617.7; and 631.7 were found in fractions 26; 27; and 28.

HPLC separation of the products of the investigated *Brevibacillus* strains were initiated, as outlined under Methods. The obtained results were summarized in Table 3. The yet unknown product family detected at *m/z* = 984.6; 1002.6; and 1020.6 Da represents the most hydrophilic compounds appearing in fractions 12–16. The brevipentins occur in fractions 26–29. They were obtained in highly purified form, as demonstrated in Figure 11. The tyrocidine family comprising numerous structural variants can be found in fractions 30 to 40. A few of them strongly overlap and need further separation. They superpose the marthiapeptide found in fractions 32–34. The most hydrophobic products are the gramicidins. Here the C15-variants appear in fractions 37–41, while C17-gramicidins were found at the end of the gradient in fractions 41–44. The HPLC-experiments form the basis to provide the products of the investigated *Brevibacillus* strains in preparative scale to perform biocontrol studies with crop plants on the molecular level.

**Table 3:** HPLC of surface extracts of *Brevibacillus* endophytes isolated from crop plants in Vietnam.

Fraction	Mass peaks/compounds *m/z*
(A) All isolated strains produce tyrocidines as the main peptide product	
30–40	Tyrocidines
(B) *Brevibacillus brevis* and porteri strains; novel genomospecies	
12–16	Unknown compounds
	984.6/1002.6/1020.6
26–29	Brevipentins
	603.3/617.3/631.3/645.3
(C) *Brevibacillus brevis*	
32–34	Marthiapeptide
	666.3/688.3/704.3
(D) *Brevibacillus parabrevis* HD3.3A and HD1.4A produce C15-gramicidins	
37–41	C15-gramicidins
	1882.0/1904.1/1920.0
(E) *Brevibacillus* sp. HB2.2 and DP1.3A produce C17-gramicidins	
41–44	C17-gramicidins
	2042.9/2064.9/2080.9

## Discussion

In this report we initiated an extensive mass spectrometric study of the biosynthetic potential of 11 *Brevibacillus* strains isolated from crop plants in Vietnam which revealed as potent producers of bioactive compounds. Four representative test strains, one of each *Brevibacillus* species, i. e. MS2.2 (*B. brevis*); HD3.3A (*B. parabrevis*); HB2.2 (*Brevibacillus* sp.) and HB1.1 (*B. porteri*) were investigated in detail by MALDI-TOF MS. The obtained results were shown in Figures 2–5, 9. Their biosynthetic potential was corroborated by that of the corresponding type strains and discussed in a wider scope performing antiSMASH 6.0-genome mining studies with *Brevibacillus* strain genome sequences deposited in the NCBI database. The results of these studies are summarized in Table 1.

Our efforts were focused on the mass spectrometric detection and structural characterization of nonribosomal peptides and polyketides as well as NRPS-PKS hybrids produced by the 11 *Brevibacillus* strains from Vietnam. All nonribosomal peptides formed by these organisms were identified by MALDI-TOF MS, while the polyketide macrobrevin as well as aurantinin- and zwittermycin A-like compounds implied by antiSMASH genome mining could not yet be observed. Here it remains to be clarified, whether the polyketide biosynthetic genes were not expressed, or we have to improve our cultivation and detection procedures to find these products. The tyrocidines revealed as the dominating products of all 11 Vietnam strains. The well-known C15-gramicidins (Tang et al., 1992; Govaerts et al., 2001; Chitta and Gross, 2004; Kessler et al., 2004) were formed by the *B. parabrevis* strains HD3.3A and HD 1.4A, while the novel genomospecies HB2.2 and DP1.3A produced C17-gramicidins recently reported under the name britacidins by Hansen et al. (2020). The highlight of our mass spectrometric detection and structure analysis by LIFT-MALDI-TOF/TOF fragment analysis was the discovery of a novel class of pentapeptides which we designate brevipentins. They were found as products of *B. schisleri*; *B. brevis* and *B. porteri*, but not of *B. parabrevis*.

Evaluation of the biosynthetic potential of these *Brevibacillus* species was performed by combination of our mass spectrometric approach with phylogenetic analysis and extensive genome mining studies by comparison with the corresponding type strains and strains whose genome sequences are deposited in the NCBI data base. The accession numbers of the genomes of all these organisms for tree construction are listed in Supplementary Table S1. In accordance with our previous phylogenomic study (Jähne et al., 2023) the phylogenetic tree presented in Figure 1 is dissected into three clades A-C. Clade A comprises numerous *B. laterosporus* and *B. halotolerans* strains which produce a great arsenal of attractive compounds, such as the lipopeptides bogorols; laterocidin; brevicidine, laterosporulin and tauramide as well as the polyketide basiliskamide, for example (Yang and Yousef, 2018; Decker et al., 2022). In contrast, most of the *Brevibacillus* strains in clade B are of rather low biosynthetic productivity. *B. parabrevis* fits into this clade. It shows a relatively limited biosynthetic capacity. It only produces tyrocidines and the well-known C15-gramicidins, but no polyketide compound. The Vietnam strains HD3.3A and HD1.4A were found in close vicinity with the *B. parabrevis* type strain DSM 8376 (see grey box in the upper part of clade B). The other Vietnam strains investigated in our work are located in clade C which contains some of the most productive *Brevibacillus* species, such as *B. schisleri*; *B. brevis*; *B. formosus*; and *B. porteri* which are potent producers of important secondary metabolites. As shown previously (Jähne et al., 2023) very clear results have been obtained for *B. porteri*. Here strains HB1.1; HB1.2; and HB1.4B which are closely related to the *B. porteri* type strain B-41110^T^ cluster in the green box of clade C. *B. porteri* strains form tyrocidines and brevipentins. MS2.2 is closely related to *B. brevis* strains DZQ7 and Leaf 182. It was therefore attributed to the species *B. brevis* (see Table 1).

**Figure 12:**
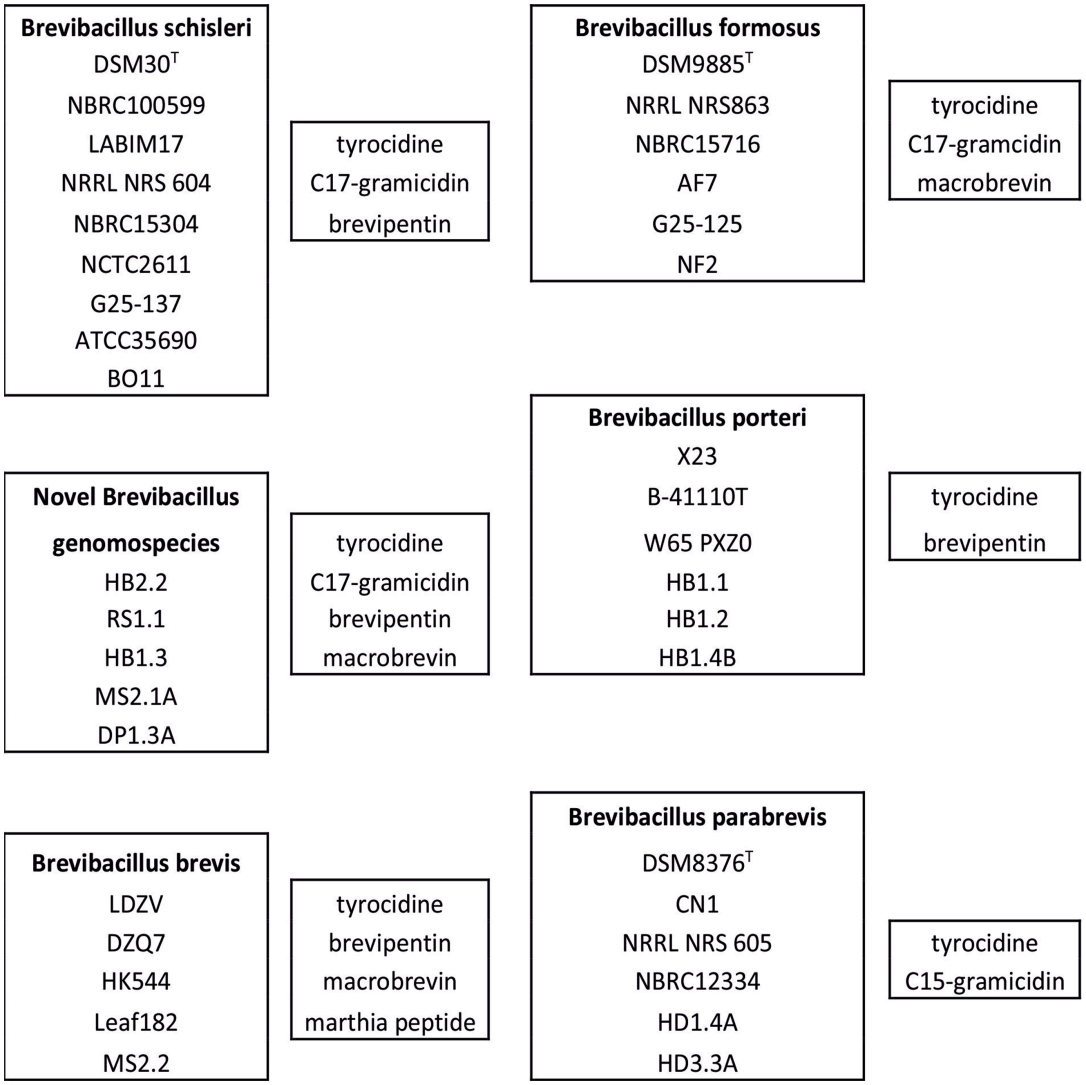
Typing of *Brevibacillus schisleri*; *brevis*; *parabrevis*; *formosus*; and *porteri* strains as well as of the novel genomospecies according to their bioactive products. From the MALDI-TOF-mass spectra in Figures 2C, 4D, 5C it is apparent that *B. brevis* MS2.2; *Brevibacillus* sp. HB2.2 and *B. porteri* HB1.1 produce a yet unknown family of compounds with molecular masses of *m/z* = 603.3; 617.3; 631.3; and 645.3 which have not been described in the literature so far.

Species relationships between the *Brevibacillus* strains according to their biosynthetic capacity is demonstrated in Figure 12 which shows the specific products of *B. schisleri*; *B. brevis*; *B. formosus*; *B. porteri*; and *B. parabrevis*. The corresponding *Brevibacillus* strains are apparent from the five boxes arranged in Figure 12. Every *Brevibacillus* species is distinguished by a specific pattern of secondary metabolites. These results demonstrate that *Brevibacillus* sp. can be classified by combination of the mass spectrometrically detected secondary metabolites with genome mining data. The five novel genomospecies in box 2 form an own group of *Brevibacillus* strains as far as the formation of secondary metabolites is concerned. They produce tyrocidines; C17-gramicidins, brevipentins and the polyketide macrobrevin. They are distributed among clade C in the phylogenetic tree in Figure 1 at different locations. Strains RS1.1; HB2.2 and HB1.3 were found to overlap with the *B. schisleri* region indicating genomic relationship with this species. The other two isolates MS2.1A and DP1.3A are located in close vicinity of the *Brevibacillus formosus* strains around the type strain DSM 9885^T^ (green box in Figure 1). From this feature we imply that these two isolates from Vietnam may be related to *B. formosus*.

In essence, an important statement of our work is that rapid typing of *Brevibacillus* spp. is possible with high certainty by generation of a mass spectrum of the produced metabolite products in combination with genome mining data thus efficiently assisting genomic classification. On the basis of our results obtained for *Brevibacillus* spp. here we introduce the product evaluation method for taxonomic classification of *Bacillus* spp. which yields results of high accuracy as demonstrated from Table 1 and Figure 12. Under most advantageous conditions significant taxonomic evaluation of a strain may be possible only by record of a mass spectrum of high resolution which can be achieved in a minimum of time. We have already successfully applied this method in a previous report on the *B. subtilis* complex (Mülner et al., 2020). According to this method we suggest the assignment for the following *Brevibacillus* strains indicated as “inconclusive” in clade A: Strains LABIM 17; NBRC 100599; G25-137; and ATCC 35690 we attribute to *B. schisleri*, while strains BO11; DZQ7; and HK 544 we assign as *B. brevis*.

Aims of the future research of our mass spectrometric product screening will be the detection and characterization of polyketide, lanthipeptide and bacteriocin products of the *Brevibacillus* isolates from Vietnam and efforts to investigate the biocontrol effects of their secondary metabolite products on the molecular level.

## Conclusion

The biosynthetic potential of 11 *Brevibacillus* strains isolated from crop plants in Vietnam was studied by a combination of MALDI-TOF MS, extensive genome mining using antiSMASH 6.0 and phylogenetic analysis. These isolates belong to the *Brevibacillus* species *brevis*; *parabrevis*; and *porteri*. In addition, five novel *Brevibacillus* genomospecies were characterized. Each of these species is distinguished by a specific pattern of secondary metabolites. Our work was focussed on the detection and structural characterization of nonribosomal peptides using LIFT-MALDI-TOF/TOF fragment analysis. All these organisms produced the tyrocidines as the main products. A novel family of pentapeptides was discovered which we designate brevipentins. The biosynthetic capacity of these species was studied in a wider context including the so far genome sequenced *Brevibacillus* strains deposited in the NCBI genome data base. Our findings are of fundamental importance demonstrating that rapid typing of *Brevibacillus* species is possible by exploration of their bioactive compounds supporting genomic classification of such organisms.

## Data availability statement

The raw data supporting the conclusions of this article will be made available by the authors, without undue reservation.

## Author contributions

JJ: Data curation, Investigation, Methodology, Writing – original draft. SH: Data curation, Investigation, Methodology, Writing – review & editing. JD: Data curation, Investigation, Methodology, Writing – review & editing. PL: Funding acquisition, Investigation, Methodology, Project administration, Supervision, Writing – original draft. LT: Funding acquisition, Investigation, Methodology, Writing – review & editing. RB: Funding acquisition, Investigation, Methodology, Project administration, Writing – review & editing. JV: Conceptualization, Funding acquisition, Investigation, Methodology, Project administration, Supervision, Writing – original draft.

## Footnotes

^1^ https://help.ezbiiocloud.net/ubcg-gene-set/

^2^ http://hmmer.org/
