# Supplementary material for: Investigation of the potential of Brevibacillus spp. for the biosynthesis of nonribosomally produced bioactive compounds by combination of genome mining with MALDI-TOF mass spectrometry
Source: Front Microbiol. 2023 Dec 14;14:1286565. doi: 10.3389/fmicb.2023.1286565 (PMC10753013; doi:10.3389/fmicb.2023.1286565)
Supplement: Supplementary file 1 [file Data_Sheet_1.docx]

**Supplementary Material**


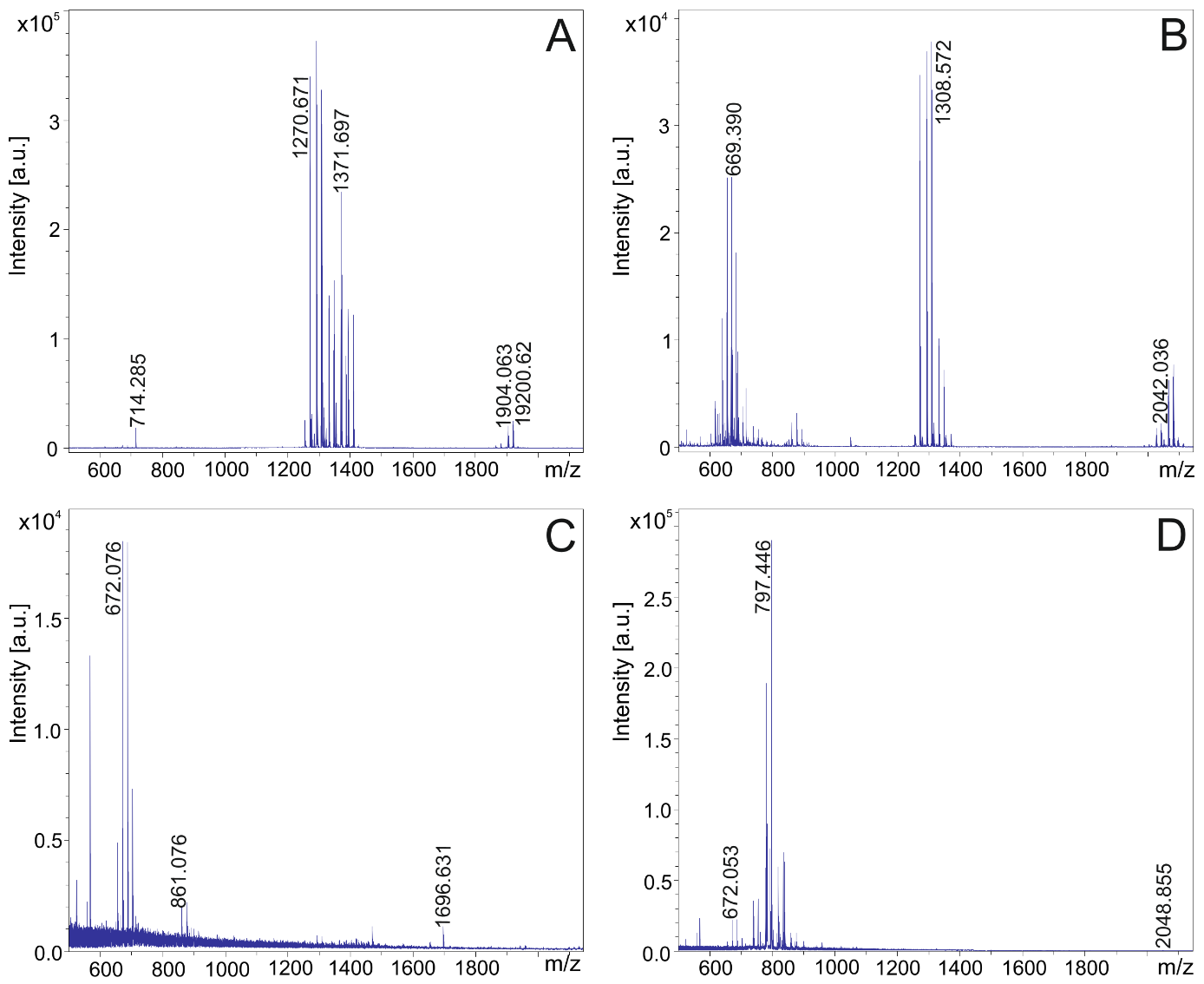


**Figure S1**

Localization of the bioactive compounds produced by *Brevibacillus parabrevis* (**A** and **C**) and for *Brevibacillus sp.* HB2.2 (**B** and **D**). (**A** and **C**) MALDI-TOF mass spectra of a surface extract of strain HD3.3A grown on agar plates using the Landy medium for 48 h (**A**) and of a culture filtrate of this strain cultivated in this medium for 48 h (**C**). (**B** and **D**) MALDI-TOF mass spectra of a surface extract of strain HB2.2 (**B**) and of a culture filtrate of this strain cultivated in the Landy medium for 48 h (**D**).


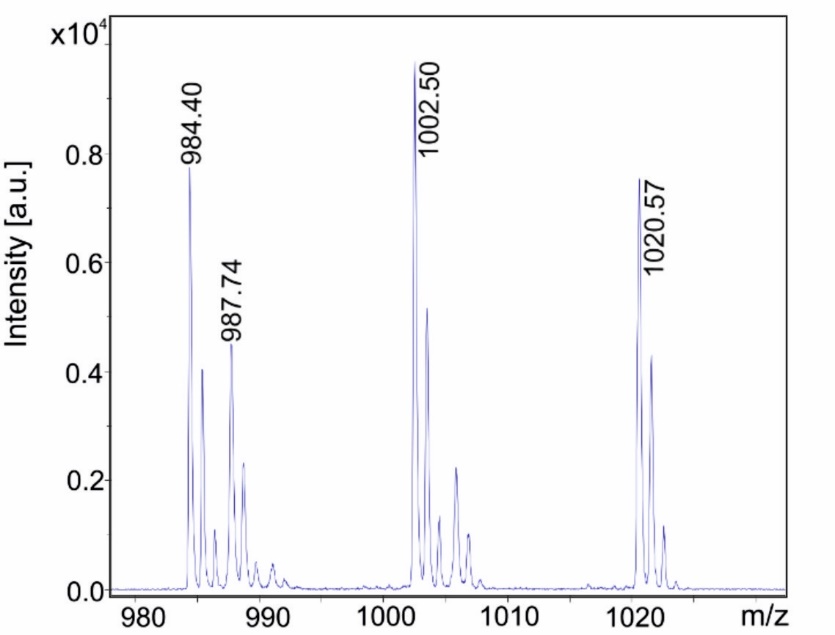


Figure S2. Unknown compounds with mass numbers of 984.4; 1002.5 and 1020.6 Da produced by *Brevibacillus sp*. HB2.2.


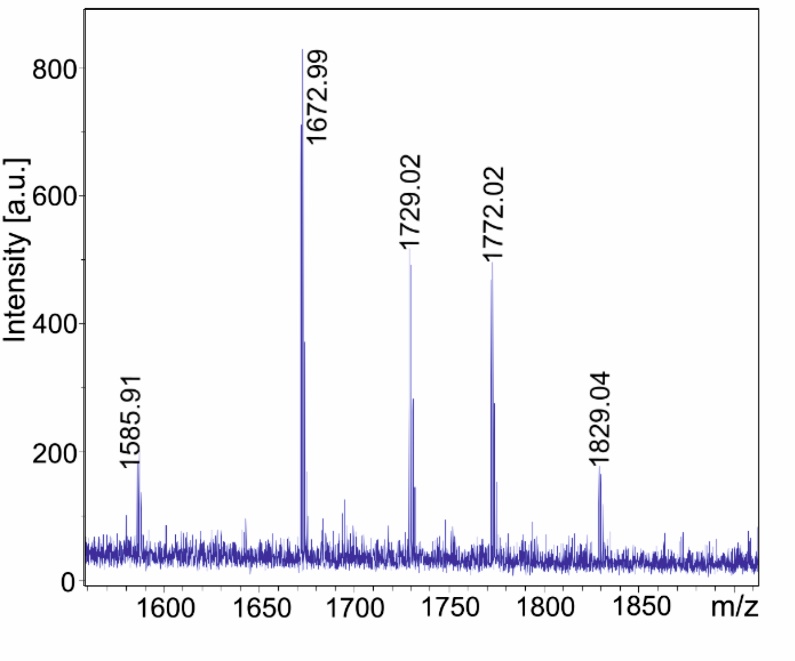


Figure S3 Unnown compounds with mass numbers of 1673.0; 1729.0; 1772.0 and 1829.0 Da produced by *B. porteri* HB1.1.

**Table S1** *Brevibacillus* strains used for construction of the phylogenetic tree in Fig. 1

Outgroup: Paenibacillus polymyxa DSM 36 (GCA_015710975.1)

**a.) Brevibacillus strains from Vietnam (n=10)**

| *Brevibacillus* | *sp.* | HB2.2 | JABSUV000000000 |
| --- | --- | --- | --- |
| *Brevibacillus* | *sp.* | RS1.1 | JABSVA000000000 |
| *Brevibacillus* | *sp.* | DP1.3A | JABSUU000000000 |
| *Brevibacillus* | *sp.* | HB1.3 | JABMIT000000000 |
| *Brevibacillus* | *sp.* | M2.1A | JABSUY000000000 |
| *Brevibacillus* | *sp.* | HD1.4A | JABSUW000000000 |
| *Brevibacillus* | *sp.* | HD3.3A | JABSUX000000000 |
| *Brevibacillus* | *sp.* | HB1.1 | JABMIV000000000 |
| *Brevibacillus* | *sp.* | HB1.2 | JABMIU000000000 |
| *Brevibacillus* | *sp.* | HB1.4B | JABSUT000000000 |

**b.) Brevibacillus type strains (n=10)**

| *Brevibacillus* | *parabrevis* | B3 | GCA_022701015.1 |
| --- | --- | --- | --- |
| *Brevibacillus* | *formosus* | DSM 9885 | GCA_001012775.1 |
| *Brevibacillus* | *formosus* | NRRL NRS-863 | GCA_003013405.1 |
| *Brevibacillus* | *parabrevis* | NBRC 12334 | GCA_006539065.1 |
| *Brevibacillus* | *brevis* | DSM 30 | GCA_003385915.1 |
| *Brevibacillus* | *brevis* | NBRC 15304 | GCA_006539845.1 |
| *Brevibacillus* | *porteri* | NRRL B-41110 | GCA_003013475.1 |
| *Aneurinibacillus* | *migulans* | DSM 2895 (T) | GCA_001274715.1 |
| *Brevibacillus* | *fortis* | NRRL NRS-1210 | GCA_003013395.1 |
| *Brevibacillus* | *agri* | NRS-1219 (T), DSM 6348 | GCA_003710885.1 |

**c. ) Brevibacillus brevis (n=15)**

| *Brevibacillus* | *brevis* | X23 | GCA_000296715.2 |
| --- | --- | --- | --- |
| *Brevibacillus* | *brevis* | FJAT-0809-GLX | GCA_000346255.1 |
| *Brevibacillus* | *brevis* | NBRC 100599 | GCA_000010165.1 |
| *Brevibacillus* | *brevis* | DZQ7 | GCA_001039275.2 |
| *Brevibacillus* | *brevis* | HK544 | GCA_007725005.1 |
| *Brevibacillus* | *brevis* | LABIM17 | GCA_021401445.1 |
| *Brevibacillus* | *brevis* | BO11 | GCA_022026395.1 |
| *Brevibacillus* | *brevis* | NCTC 2611 | GCA_900637055.1 |
| *Brevibacillus* | *brevis* | NRRL NRS-604 | GCA_003012835.1 |
| *Brevibacillus* | *brevis* | Ag35 | GCA_014526365.1 |
| *Brevibacillus* | *brevis* | NBRC 110488 | GCA_001748185.1 |
| *Brevibacillus* | *brevis* | I2-B3 | GCA_019749035.1 |
| *Brevibacillus* | *brevis* | G25-137 | GCA_015912885.1 |
| *Brevibacillus* | *brevis* | ATCC 35690 | GCA_002161835.1 |
| *Brevibacillus* | *brevis* | GZDF3.1 | GCA_001649505.1 |

**d.) Brevibacillus formosus (n=4)**

| *Brevibacillus* | *formosus* | NF2 | GCA_002215075.1 |
| --- | --- | --- | --- |
| *Brevibacillus* | *formosus* | NBRC 15716 | GCA_006540185.1 |
| *Brevibacillus* | *formosus* | G25-125 | GCA_015845835.1 |
| *Brevibacillus* | *formosus* | AF7 | GCA_019400865.1 |

**e.) Brevibacillus parabrevis (n=3)**

| *Brevibacillus* | *parabrevis* | 179-C7.2 HS | GCA_019037975.1 |
| --- | --- | --- | --- |
| *Brevibacillus* | *parabrevis* | CN1 | GCA_001619605.1 |
| *Brevibacillus* | *parabrevis* | NRRL NRS 605 | GCA_003710905.1 |

**f.) Brevibacillus laterosporus (n=31)**

| *Brevibacillus* | *laterosporus* | LMG 15441 | GCA_000219535.3 |
| --- | --- | --- | --- |
| *Brevibacillus* | *laterosporus* | PE36 | GCA_000472325.2 |
| *Brevibacillus* | *laterosporus* | DSM 25 | GCA_000374385.1 |
| *Brevibacillus* | *laterosporus* | GI-9 | GCA_000237005.2 |
| *Brevibacillus* | *laterosporus* | E7593-50 | GCA_003590075.1 |
| *Brevibacillus* | *laterosporus* | 1821 | GCA_007833815.1 |
| *Brevibacillus* | *laterosporus* | ZQ2 | GCA_002865525.1 |
| *Brevibacillus* | *laterosporus* | VKPM_B-9406 | GCA_024741985.1 |
| *Brevibacillus* | *laterosporus* | VKPM_B_13244 | GCA_024742035.1 |
| *Brevibacillus* | *laterosporus* | Uniss_18 | GCA_001696705.1 |
| *Brevibacillus* | *laterosporus* | Lak 1210 | GCA_002412145.1 |
| *Brevibacillus* | *laterosporus* | VKPM_B_10531 | GCA_024742055.1 |
| *Brevibacillus* | *laterosporus* | VKPM_B_13242 | GCA_024742015.1 |
| *Brevibacillus* | *laterosporus* | BGSP7 | GCA_002927075.1 |
| *Brevibacillus* | *laterosporus* | ACRRF | GCA_022346405.1 |
| *Brevibacillus* | *laterosporus* | OSY-I1 | GCA_002259955.1 |
| *Brevibacillus* | *laterosporus* | G25-128 | GCA_015845895.1 |
| *Brevibacillus* | *laterosporus* | BON707 | GCA_003594765.1 |
| *Brevibacillus* | *laterosporus* | G25-130 | GCA_015845885.1 |
| *Brevibacillus* | *laterosporus* | K75 | GCA_012328875.1 |
| *Brevibacillus* | *laterosporus* | MG64 | GCA_003265735.1 |
| *Brevibacillus* | *laterosporus* | BGSP11 | GCA_002926995.1 |
| *Brevibacillus* | *laterosporus* | VKPM_B_13247 | GCA_024741975.1 |
| *Brevibacillus* | *laterosporus* | G25-131 | GCA_015845945.1 |
| *Brevibacillus* | *laterosporus* | BGSP9 | GCA_002927085.1 |
| *Brevibacillus* | *laterosporus* | NRS590 | GCA_006438615.1 |
| *Brevibacillus* | *laterosporus* | Rsp | GCA_006438655.1 |
| *Brevibacillus* | *laterosporus* | CCEB342 | GCA_006438635.1 |
| *Brevibacillus* | *laterosporus* | G25-129 | GCA_015845875.1 |
| *Brevibacillus* | *laterosporus* | SAM19 | GCA_016904905.1 |

**g.) Weitere Brevibacillus Spezies (n=9)**

| *Brevibacillus* | *centrosporus* | NRS 664 (T) | GCA_003710815.1 |
| --- | --- | --- | --- |
| *Brevibacillus* | *invocatus* | JCM 12215 | GCA_003710915.1 |
| *Brevibacillus* | *gelatini* | DSM 100115 | GCA_003710935.1 |
| *Brevibacillus* | *nitrificans* | JCM 15774 | GCA_003710965.1 |
| *Brevibacillus* | *panacihumi* | JCM 15085 | GCA_003710985.1 |
| *Brevibacillus* | *choshinensis* | DSM 8552 | GCA_001420695.1 |
| *Brevibacillus* | *massiliensis* | phR/DSM 25447 | GCA_000311785.1 |
| *Brevibacillus* | *panacihumi* | W25 | GCA_000503775.1 |
| *Brevibacillus* | *agri* | DSM 6348 (T) | GCA_004117055.1 |

**Summe: 82 Genome**

**h.) Genome weiterer Spezies, zusätzlich in die Analysen aufgenommen (n=27):**

| *Brevibacillus* | *composti* | FJAT-54423 (T) | GCA_016406105.1 |
| --- | --- | --- | --- |
| *Brevibacillus* | *composti* | FJAT-54424 | GCA_018228725.1 |
| *Brevibacillus* | *marinus* | SCSIO 07484 (T) | GCA_003963515.1 |
| *Brevibacillus* | *antibioticus* | TGS2-1 | GCA_005217615.1 |
| *Brevibacillus* | *fulvus* | DSM 25523 | GCA_016908525.1 |
| *Brevibacillus* | *fluminis* | JCM 15716 (T), DSM 23904 | GCA_003710825.1 |
| *Brevibacillus* | *migulae* | CFH S0501 (T), DSM 29940 | GCA_004521915.1 |
| *Brevibacillus* | *borstelensis* | NRRL NRS 818 (T), DSM 6347 | GCA_003710865.1 |
| *Brevibacillus* | *borstelensis* | AK1 | GCA_000353565.1 |
| *Brevibacillus* | *borstelensis* | NBRC 15714 | GCA_006540165.1 |
| *Brevibacillus* | *borstelensis* | MSL 140.1 | GCA_023712885.1 |
| *Brevibacillus* | *borstelensis* | cifa_chp40 | GCA_000738785.1 |
| *Brevibacillus* | *borstelensis* | 3096-7 | GCA_000612185.1 |
| *Brevibacillus* | *borstelensis* | GP-1-2017 | GCA_013140865.1 |
| *Brevibacillus* | *borstelensis* | M42 | GCA_020640885.1 |
| *Brevibacillus* | *reuszeri* | DSM 9887 (T) | GCA_001187725.1 |
| *Brevibacillus* | *reuszeri* | J31TS6 | GCA_018333155.1 |
| *Brevibacillus* | *reuszeri* | NBRC 15719 | GCA_006540225.1 |
| *Brevibacillus* | *halotolerans* | s-14 | GCA_013867635.1 |
| *Brevibacillus* | *halotolerans* | J5TS2 | GCA_018333135.1 |
| *Brevibacillus* | *thermoruber* | PM1 | GCA_000744635.1 |
| *Brevibacillus* | *thermoruber* | 423 | GCA_000454065.1 |
| *Brevibacillus* | *centrosporus* | NBRC 15540 | GCA_006540045.1 |
| *Brevibacillus* | *centrosporus* | OK042 | GCA_900114075.1 |
| *Brevibacillus* | *agri* | BF-47S | GCA_022214745.1 |
| *Brevibacillus* | *agri* | NBRC 15538 | GCA_006540025.1 |
| *Brevibacillus* | *agri* | G25-57 | GCA_015844595.1 |
